# Commentary: National and international collaboration for instrumentation in health care laboratories

**DOI:** 10.1155/S1463924681000163

**Published:** 1981

**Authors:** F. L. Mitchell


### Commentary

### National and international collaboration for instrumentation in health care laboratories

The professions connected with health care laboratories have come to rely heavily on complex instruments and it is therefore not surprising that much has been done over the last two decades to rationalise design and efficiency and stimulate collaboration nationally and internationally between those practising within the profession itself, and those in the associated production industry.

At the personal level, much has been achieved by inventors in the profession passing on their ideas to industry, and many helpful consultancies have been arranged, particularly in the USA. However, with the evolution of an associated technically highly advanced industry, much of the innovation has passed to industry itself. This was to be expected and most industrial companies have favourite health care laboratories where they can try out new products and principles.

National societies in many countries, mainly in clinical chemistry, soon saw the need to form specialist committees in instrumentation and/or automation and data processing, and much experience has been gained by these groups in the operation and design of evaluation protocols with feedback to companies of much valuable advice.

Some 13 years ago in the USA a few far-sighted individuals appreciated the necessity for collaboration between the three major groups involved in laboratory medicine (governmental, industrial and professional) if standards were to be improved, and the National Committee for Clinical Laboratory Standards (NCCLS) was formed. It was agreed that without good instruments there was little chance of improving the quality of work and its Committee on Instrumentation has always been one of the most active area committees of NCCLS.

Written standards for the many different aspects of medical laboratory work need to be specified after very careful deliberation, and NCCLS took on the role of producing these in ever-increasing quantity until now they are appearing fairly regularly at the rate of three per month. They are so-called "parametric" in nature since they do not normally for example set limits as such, but rather state how such limits should be specified and attained; methods of procedure are carefully described. The most important NCCLS standards in the field of instrumentation are as follows; they may be obtained from the NCCLS, 771 E. Lancaster Avenue, Villanova, PA 19085, USA.

- ASI-1 Preparation of Manuals for Installation, Operation, and Repair of Laboratory Instruments (Second Edition)
- ASI-2 Standard for Temperature Calibration of Water Baths, Instruments, and Temperature Sensors
- ASI-3 Standard for Determining Spectrophotometric Performance Criteria
- PSI-4 Guideline to the Selection of Accuracy Classes of Thermistor Thermometers and the Verification of their Accuracy
- TSI-5 Power Requirements for Clinical Laboratory Instruments and for Laboratory Power Lines
- TSI-6 Guidelines for Service of Clinical Laboratory Instruments
- I7-P Standard for Photometric Systems for Measuring Reaction Rates.

Understandably the standards produced by NCCLS had immediate popular support since the rules governing their agreement ensure consensus between all interested parties. However, since no other body was active in producing similar documents, and in the absence of any international agreements or documentation of any kind, industry began to accept NCCLS standards for international use.

The American Committee was not entirely happy with the role of producing the world's standards and covering single-handed the large amount of work and finance involved; nor, with respect, were many European countries entirely happy at having to accept standards when they had had little or no say in their drafting. Hence in 1979 another similar organisation came into being - the European Committee for Clinical Laboratory Standards (ECCLS), and it is interesting that considering all the work urgently required, a decision was made for the first major task to be the development and operational establishment in Europe of a protocol for the evaluation of automatic analysers. This is approaching final draft stage.

As instruments have become increasingly complex and expensive, a correspondingly increasing amount of time and expense has had to be expended by both industry and the profession in their testing and evaluation in an ever-expanding number of laboratories and countries. Few customers would accept evaluation work done in other laboratories, often in other countries, with the result that this work has had to be replicated on an alarming scale. The United Kingdom with its government-run National Health Service was the first country to organise a national evaluation scheme but recently professional organisations in other countries (notably Australia, France, Germany and some Scandinavian countries) have started schemes of their own. In 1975, an effort was made to interest the European Community Bureau of Reference in Brussels in a scheme whereby the evaluation of instruments for use in health care laboratories might be centralised for the countries of the European Community. This approach was not successful at the time because it was appreciated that if such a scheme were accepted, there would be good reason to accept similar schemes for domestic appliances such as dish washers and washing machines and major undertakings of this nature seemed neither practical nor desirable.

With the evolution of ECCLS however it seemed that if a testing protocol could be brought into being whereby an instrument could for example be tested in three laboratories in at least two different countries, then the results of such an evaluation might be acceptable throughout all countries with membership of ECCLS. It is with this ideal in mind that the current evaluation protocol is being produced and a possible scheme for its application is being devised.

In clinical chemistry, the International Federation for Clinical Chemistry (IFCC) exists to forge international links between clinical chemists in all countries in scientific, professional and organisational matters. The Federation carries out its scientific work mostly through its expert panels which are set up from time to time to deal with specific problem areas. In 1975 a decision was made to form an Expert Panel on Instrumentation which has now been active throughout the world for some six years. From its inception it became evident that to be of maximum value this Panel should differ somewhat from the others in that as well as studying instrumentation problems in depth it was necessary to foster international relationships between all those involved in the design, manufacture and use of instruments and in addition, stimulate the development of systems for the promulgation of information and education in instrumentation generally.

With these aims in view, the Committee incorporated into its membership representatives from some 60 instrument companies, in addition to its professional Associate Members from 42 countries.

At the first of the open meetings which the Panel has held at all the recent major congresses in clinical biochemistry, it was stressed that an important requirement worldwide was for guidelines for instrument manufacturers when describing new instruments. So often in advertisements or at exhibition booths, vital information required by a potential customer is either not given or not available. The production of documents to satisfy this need was given high priority and several have either already appeared in the press or are in developement, covering automatic analysers (*J. Clin. Chem. Clin. Biochem.* **18**, 947, 1980), spectrometers (*Clin. Chim. Acta* **103**, 249F, 1980), atomic absorption and flame emision spectrometers and nephelometers.

In 1977 it was suggested by the Panel's Associate Member in New Zealand that the Panel might be able to help isolated clinical chemists in particular, to choose instruments best suited to their home situations from the often bewildering selection available. A symposium entitled "Decision Criteria for Selecting Analytical Instruments" was organised during the Third European Congress of Clinical Chemistry held at Brighton, England, in 1979, and repeated for the First South-East Asian and Pacific Congress in Singapore also in 1979. It was suggested in Singapore that the main lectures given during the Symposium should be made available, together with slides, for IFCC members to present to audiences where appropriate. Sets of these papers and slides have been generously prepared by EI Du Pont de Nemours and Co and are available from any Panel members or from the Division of Clinical Chemistry, Clinical Research Centre, Harrow, Middlesex, England.

At the 1980 Biochemische Analytik in Munich, two meetings were held to discuss ways of standardising computer interfaces and machine-readable patient identification. As a result, a decision was made to set up small specialist groups within Europe to monitor the positions in the two areas and do all possible to expedite some degree of standardisation.

The supply and maintenance of instruments for developing countries remains a continual worry. Conditions for most of the world's populations are not suitable for the satisfactory operation of instruments widely used in the developed world and attention needs to be paid to the design of equipment which will operate in the absence of good electricity supplies, good climatic conditions, high technological education of operators and regular and good maintenance facilities. For many and varied reasons it has proved to be very difficult to cover these requirements but the Panel in collaboration with the World Health Organisation has strenuously endeavoured to deal with the problem.

From April 19-22, 1982, the Sociedad Espanola de Quimica Clinica is organising an International Congress on Automation in the Clinical Laboratory. The Society asked the Panel to organise the scientific content of the Congress and the programme is now complete. This will be the first major international congress on automation and the three plenary lectures will cover automation in clinical chemistry, microbiology and haematology. These will be followed by symposia on solid phase chemistry, kinetic measurements, immobilised enzymes, instruments for developing countries, cost analysis, computer interfaces and patient identification, new technology allowing pathology near the patient, and nomenclature for automated analysis. The address of the Congress organisers is: Apartado de Correos 543, Barcelona, Spain.

It will be apparent from the above that the last two decades have seen the build up of a considerable amount of national and international activity covering instrumentation in the clinical laboratory. The size of the associated multibillion dollar industry can best be judged by noting the extent of the exhibitions which now accompany international congresses. The pattern which seems to be evolving for the future is one whereby the activities of individuals in the profession are channeled through national professional societies, and industrial companies also interface with the profession through such societies. On a regional basis the involvement of governments, industry and the professions in now happily co-ordinated in the areas covered by NCCLS and ECCLS. It is hoped that in the future a third such organisation might appear in the Far East and if NCCLS could expand to cover the Americas most countries could be included in the network. The role of international organisations such as the IFCC Expert Panel on Instrumentation would then be more of a co-ordinating nature. This is already proving to be a necessary function, since conditions in different parts of the world often necessitate very different approaches to problems.

*F. L. Mitchell*

### UK conferences and courses

The multidisciplinary approach to education in automated chemistry was highlighted in the Commentary in the last issue of The Journal (page 3). Two significant developments were referred to; the new Department of Instrumentation and Analytical Science at UMIST under Prof. Gordon Kirkbright, and the appointment of Prof. D. Betteridge to a personal chair of Analytical Sciences at Swansea. While the importance of these developments cannot be overestimated we must not lose sight of the very real need for "further" education in automated chemistry. In the UK in 1981 this need is being met by a residential course and two conferences specifically aimed at bringing together the various chemical disciplines to discuss their common automation needs.

When qualified staff have not had the opportunity to study automation in their formal training, their employers can compensate by sending them on the Swansea Summer School of Automatic Chemical Analysis being held at The University College of Swansea from 5th to 10th July. The theme for this year's Summer School is "Computers in Automation and Laboratory Management". The course offers a series of lectures by outstanding world authorities on laboratory automation as well as an intensive programme of tutorials and practical sessions in which there will be the chance to obtain hands-on experience with the latest instrumentation from leading manufacturers.

Amongst the lecturers will be Dr. R.W. Arndt (Switzerland), Prof. D. Betteridge (Swansea), Dr. A. Carrick (Manchester), Dr. D.R. Deans (Middlesbrough), Dr. G. Horlick (Canada), Prof. L. Massart (Belgium), Prof. H.L. Pardue (USA), and Dr. K. Stewart (USA). Full details on this course can be obtained from Prof. D. Betteridge, Department of Chemistry, University College of Swansea, Swansea, SA2 8PP.

A symposium on Automated Analysis is to be held at the University of Stirling on the 10th and 11th September; this is being organised jointly by a number of groups within the Royal Society of Chemistry. The speakers will include Prof. D. Betteridge, Dr. J.C. Berridge, Dr. A. Carrick, Dr. A.S. McHelland, Dr. D.A. Newton, Dr. R.M. Smith, Dr. P.B. Stockwell and Dr. R.L. Tranter. Their topics will cover the influence of microprocessors and microcomputers on automation, developments in hardware, interfacing microcomputers to analytical equipment, and data handling. Further information is available from Dr. C.J. Jackson, Health & Safety Executive, 403 Edgware Road, London, NW2 6LN.

In the following week (15th and 16th September) a conference in London will appeal to industrially based analytical chemists. Analysis 81 this year is entitled "Micro and Mini Computers in the Laboratory". Again there will be an intensive programme of lectures and panel discussions in which current thinking and practice will be considered by experienced users.

Review lectures at this conference will be delivered by Dr. R.F. Coleman, Dr. E.L. Dagless and Dr. A. Carrick. A detailed prospectus is available from Scientific Symposia Ltd., 33-35 Bowling Green Lane, London, EC1R 0DA, UK.

